# Translational Regulation of Plant Stress Responses: Mechanisms, Pathways, and Applications in Bioengineering

**DOI:** 10.1146/annurev-phyto-121823-032335

**Published:** 2025-06-02

**Authors:** Yezi Xiang, Xinnian Dong

**Affiliations:** 1 Department of Biology, Duke University, Durham, North Carolina, USA; 2 Howard Hughes Medical Institute, Duke University, Durham, North Carolina, USA

**Keywords:** translational regulation, stress response, mRNA stability, mRNA structure, mRNA modification, tRNA availability, ribosome biogenesis, ribosome heterogeneity

## Abstract

Understanding how organisms regulate protein translation in response to stress is vital for both fundamental biology and biotechnological innovation. However, our knowledge of this area remains limited due to the inherent complexity of the translational regulatory process. Recent advances in multiomics and single-molecule technologies now allow for an integrated analysis of the multilayered regulation of translation in plants in response to biotic and abiotic stresses. In this review, we provide essential background information for newcomers to the field and synthesize recent discoveries in stress-induced translation into the following key areas: mRNA features (cap, Kozak sequence, uAUGs and uORFs, secondary structures, modifications, alternative splicing, small RNAs), ribosomal biogenesis and heterogeneity, tRNA and codon usage, master translation regulatory factors, spatial dynamics of translation, tools for studying translation regulation, and translational engineering for crop resilience. In assembling this review, we also uncovered significant knowledge gaps that represent exciting opportunities for future research.

## 1. INTRODUCTION

Plants are constantly exposed to environmental challenges, including biotic stresses like pathogens and viruses, and abiotic stresses such as drought, heat, cold, hypoxia, and salinity. To survive and adapt, they rely on precise and dynamic control of gene expression. In this process, translational regulation, the direct control of protein production from messenger RNA (mRNA), is essential for rapidly mounting responses to stress. Despite its importance, translational regulation remains less studied than transcriptional regulation, largely due to the complexity of the process (23, 76, 171). This includes multiple interconnected layers of regulation, such as mRNA features, transfer RNA (tRNA) availabilities, translational machineries, and spatial dynamics, which may all play a critical role in plant stress responses (23). Fortuitously, recent advancements in research techniques, such as ribosome profiling (Ribo-Seq) (82, 83) and spatial omics (156), have greatly expanded our ability to study how translation is regulated under various physiological and environmental conditions.

Plants not only utilize common translational regulatory mechanisms observed in other organisms but also employ unique strategies that are adapted to their sessile lifestyle and specific stress-response needs. For instance, during pathogen attack, plants selectively translate defense mRNAs, shifting the protein production from growth to defense. Because plants do not have specialized immune cells, they must reprogram cellular activities to strike a balance between fighting infection and maintaining normal physiological activities (192, 208, 211).

These unique characteristics underscore the significance of versatility and precision in translational regulation in plants. They also offer great opportunities to uncover the underlying mechanisms to fill knowledge gaps in this last step of the central dogma of molecular biology as well as to develop bioengineering toolkits for protein production. Combined with CRISPR-Cas9 (123) and artificial intelligence (AI)-based technologies (188) for targeted modifications of endogenous genes, we can develop ways to improve crop stress tolerance and disease resistance while maintaining high plant yield.

In this review, we explore the role of translational regulation in plant stress responses and its potential for engineering disease-resistant crops. We begin with a brief overview of translational regulation in plants, emphasizing unique features compared to those conserved mechanisms. We then highlight several recent groundbreaking discoveries across different layers of regulation, such as mRNA features [e.g., upstream open reading frames (uORFs), mRNA structures, modifications, and noncanonical translation initiation components], ribosome biogenesis, tRNA availability, and translation regulators. Finally, we examine the tools available for studying translational regulation and the application potential of the new knowledge gained in agriculture.

## 2. CANONICAL TRANSLATIONAL REGULATORY MECHANISMS

The translation process in plants is evolutionarily conserved, as with other eukaryotes, and typically proceeds through four stages: initiation, elongation, termination, and ribosome recycling (18). Based on the conventional view, eukaryotic mRNAs are generally monocistronic and undergo several maturation steps before translation (10, 14). These include the addition of a 5′ m^7^GpppN cap, splicing to remove introns, and polyadenylation at the 3′ end (10). The mature mRNA is then exported from the nucleus into the cytoplasm, where translation occurs (40, 163).

**Figure 1:**
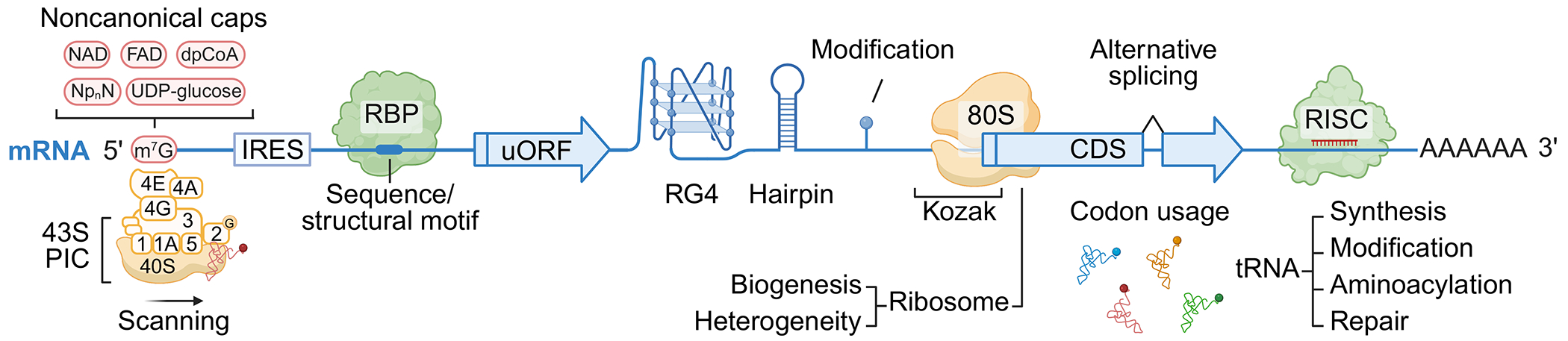
Translational regulation through messenger RNA (mRNA) features, ribosomes, and transfer RNAs (tRNAs). mRNA with 5′ m^7^GpppN cap (m^7^G) and 3′ polyadenylated tail. 1, 1A, 2, 3, 4E, 4G, 4A, and 5 are eukaryotic translation initiation factors. The initiator tRNA (Met-tRNA_i_^Met^) is attached to eIF2. Figure adapted from images created in BioRender; Xiang Y. 2025. https://BioRender.com/k14d971. Abbreviations: 40S, 40S small ribosomal subunit; 43S PIC, 43S preinitiation complex; 80S, 80S ribosome composed of the 40S small and 60S large ribosomal subunits; CDS, coding sequence/main open reading frame; dpCoA, dephospho-CoA; FAD, flavin adenine dinucleotide; G, GTP; IRES, internal ribosome entry site; Kozak, Kozak sequence; NAD, nicotinamide adenine dinucleotide; Np_n_N, dinucleotide polyphosphate; RBP, RNA binding protein; RG4, RNA G-quadruplex; RISC, RNA-induced silencing complex; UDP, uridine diphosphate; uORF, upstream open reading frame.

As shown in Figure 1, the canonical process of translation initiation begins with mRNA activation via the eukaryotic translation initiation factor complex eIF4F, which includes eIF4E (a cap-binding protein), eIF4A (an RNA helicase), and eIF4G (a scaffolding protein) (20, 60, 107, 129). Besides the 5′ cap of the mRNA recognized by eIF4E, the poly(A) tail of the mRNA, bound by the poly(A)-binding protein (PABP), may fold back to form a loop via interaction with eIF4G to enhance cap activity (20, 94). Concurrently, the 43S preinitiation complex (43S), assembled from the 40S small ribosomal subunit, eIF1, eIF1A, eIF3, eIF5, and a ternary complex formed by eIF2, GTP, and the initiator tRNA (Met-tRNA_i_^Met^), becomes associated with the activated mRNA through eIF3 interaction with eIF4G (3, 76, 171). This association forms the 48S preinitiation complex (PIC), which then starts to scan the 5′ leader sequence (5′ LS) region of the mRNA to locate the start codon (12). During scanning, ATP-dependent RNA helicase eIF4A unwinds secondary structures in the mRNA to ensure smooth progression of the process (19). Recognition of the start codon triggers the release of most eIFs and the joining of the 60S ribosomal subunit, mediated by the GTPase eIF5B, forming the 80S initiation complex (150, 189). This complex is then ready for translation.

In addition to the canonical pathway, plants employ noncanonical initiation mechanisms under specific conditions to bypass the requirement for the 5′ cap by utilizing specialized features such as internal ribosome entry sites (IRESs) (192). Noncanonical initiation is critical for translating certain viral or stress-responsive mRNAs to either hijack host translational machinery for viral replication or reprogram the plant host’s translatome from growth to defense to combat infection (186, 192). For the latter, it is often accompanied by a transient increase in the decapping activity as a mechanism to inhibit production of growth-related proteins whose translation is cap-dependent (192, 224).

During elongation, aminoacyl-tRNAs are delivered to the ribosomal aminoacyl (A) site by elongation factor 1α (eEF1A) in a GTP-dependent process (141), which involves codon–anticodon recognition and the release of eEF1A (4). The peptidyl transferase center then catalyzes the formation of a peptide bond between the growing peptide in the peptidyl (P) site and the new amino acid in the A site (96). After peptide bond formation, the GTPase elongation factor 2 (eEF2) moves the ribosome along the mRNA by translocating the tRNAs to the P and exit (E) sites, which frees the A site for the next aminoacyl-tRNA (42, 54). The deacylated tRNA in the E site then exits the ribosome, allowing the next elongation cycle until a stop codon is reached (158).

Termination begins when the ribosome encounters a stop codon (UAA, UGA, or UAG). This is mediated by a ternary complex comprising eukaryotic release factors eRF1, which recognizes the stop codon, and eRF3, which hydrolyzes GTP to catalyze the release of nascent polypeptide from the tRNA (reviewed in 74). Post-termination ribosomes are dissociated into 40S and 60S subunits and recycled by the ATPase ABCE1, triggering the release of mRNA and deacylated tRNA (151) and resetting the machinery for new rounds of translation (reviewed in 74).

Most of the translation components are conserved between plants and animals. However, plants have evolved unique factors. For example, in addition to the canonical eIF4F complex, plants contain the eIF(iso)4F complex comprising eIF(iso)4E and eIF(iso)4G [encoded by eIF(iso)4G1 and eIF(iso)4G2] (137). Furthermore, plants possess specialized translation machinery in chloroplasts (reviewed in 242) and mitochondria (reviewed in 187).

Besides these components directly involved in the translational process described above, key upstream signaling pathways, such as those controlled by target of rapamycin (TOR) (reviewed in 25, 216), sucrose non-fermenting-1-related protein kinase 1 (SnRK1) (reviewed in 22), and general control nonderepressible 2 (GCN2) (reviewed in 27, 125), play critical roles in integrating cellular and environmental signals with the translation machinery, enabling plants to appropriately respond to stress.

**Figure 2:**
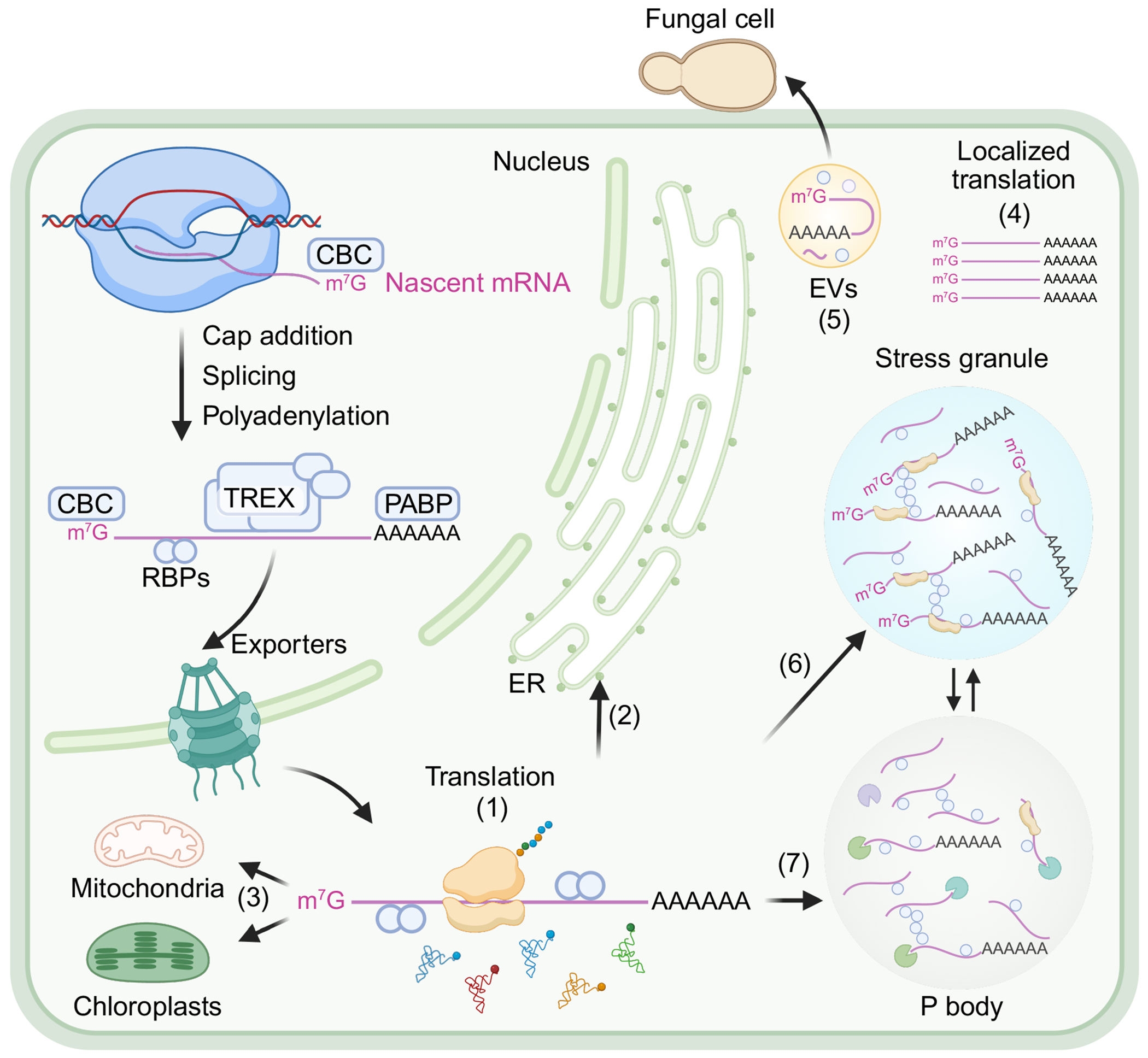
Spatial regulation of translation. During messenger RNA (mRNA) maturation, the 5′ m^7^GpppN (m^7^G) cap and 3′ tail are bound and protected by CBC (cap-binding complex) and poly(A)-binding protein (PABP), respectively. After export, mRNAs may be localized to various cellular compartments for translation, including (*a*) the cytoplasm, (*b*) the endoplasmic reticulum (ER), (*c*) organelles such as mitochondria and chloroplasts, and (*d*) specific localizations, or (*e*) packaged into extracellular vesicles (EVs) and transported to pathogens (e.g., fungal cells) for translation. During stress conditions, mRNAs may be temporarily stored in (*f*) stress granules or (*g*) P bodies or undergo degradation. Figure adapted from images created in BioRender; Xiang Y. 2025. https://BioRender.com/q93i050. Abbreviations: RBPs, RNA binding proteins; TREX, transcription-export complex.

Additionally, translation is subject to temporal and spatial regulation (Figure 2). For instance, mRNAs may be selectively transported to specific cellular compartments for localized protein synthesis (reviewed in 39, 179), or they may be sequestered in membraneless compartments such as stress granules (SGs) or P-bodies under certain conditions (reviewed in 132, 134, 149). These biomolecular condensates serve as sites for mRNA storage, degradation, or translational repression, enabling the cell to quickly undergo global reprogramming of the translatome, instead of modifying preexisting mRNA individually in response to environmental stress. On a narrower scale, mRNAs may be stored and translated only at specific stages of development (15, 238) or in response to particular signals (142), allowing the plant to fine-tune protein production in a spatially and temporally regulated manner.

In essence, translational regulation in plants is a highly conserved as well as versatile process. The complexity of this process has been reviewed in References 23, 175, 182, and 205, and here we focus on the multiple layers of regulation that enable plants to control protein synthesis in response to biotic and abiotic stress.

## 3. TRANSLATIONAL REGULATION THROUGH mRNA FEATURES

### 3.1. Cap-Independent Translation Initiation

In eukaryotes, besides the canonical cap-dependent initiation process, certain sequences or secondary structures in the mRNA can serve as IRESs to directly recruit the ribosome to initiate translation (reviewed in 109) (Figure 1). This phenomenon was first discovered and has been well-studied in viral RNAs, which lack the 5′ cap and evolved IRES sequences to hijack the host translation machinery for protein synthesis during infection (87, 109, 148). More recently, studies have shown that plants also contain cellular IRES elements, particularly in stress-responsive mRNAs (192). These IRESs serve as a mechanism to activate translation of defense proteins while growth-related protein translation is transiently dampened.

One notable example of IRES-dependent translation involves the AtLa1 protein, which binds to the 5′ LS of WUSCHEL (WUS) mRNA and facilitates IRES-dependent translation (38). Under environmental stress, AtLa1 is translocated from the nucleus to the cytoplasm, enhancing WUS mRNA translation to maintain the stem cell pool in the shoot apical meristem. However, how AtLa1 carries out this cap-independent translation has yet to be elucidated.

During plant pattern-triggered immunity (PTI), there is a general increase in the decapping activity to dampen cap-dependent translation while selectively translating mRNAs containing purine-rich motifs (R-motifs) in their 5′ LSs, which act as cellular IRESs (192). Translation from the R-motif is mediated by PABPs through preferential association with the PTI-activating eIF (iso)4G over the canonical eIF4G. This switch occurs through phosphorylation by the central PTI regulators, MITOGEN-ACTIVATED PROTEIN KINASE 3 and 6 (MPK3 and 6), leading to the inhibition of the canonical eIF4G’s activity while enhancing PABP binding to the R-motif and eIF(iso)4G-mediated translation of defense mRNAs.

Moving forward, several questions remain to be explored. First, the distribution and diversity of IRES elements in plants need further investigation. Although primary sequences of IRESs have been identified, the role of secondary structures in the 5′ LS as potential IRES elements remains unclear. Large-scale screening using cap-independent reporter systems, such as circular RNAs (29), could help identify novel IRES elements and their *trans*-acting factors. Second, from an evolutionary perspective, the origins of IRES elements in plants and their potential horizontal transfer from viruses or independent evolution need further exploration.

### 3.2. Noncanonical Caps

Recent studies have identified the presence of noncanonical caps on mRNAs across eukaryotes (reviewed in 43). These caps, derived from metabolites such as nicotinamide adenine dinucleotide (NAD), flavin adenine dinucleotide (FAD), dephospho-CoA, uridine diphosphate (UDP)-glucose, UDP-N-acetylglucosamine, and dinucleotide polyphosphates, can serve as the initiating nucleotide for transcription and play a role in influencing mRNA stability and translation (reviewed in 43, 152) (Figure 1). In plants, transcriptome-wide profiling of NAD^+^-capped mRNAs has revealed the widespread presence of this type of mRNA (44, 80, 198, 225, 231), and polysome profiling has shown that these mRNAs can be translated (198). In a recent study using SPAAC-NAD-seq (80), which offers enhanced accuracy and sensitivity in detection, NAD^+^-capped mRNAs were shown to play crucial roles in key biological processes, including responses to abiotic stresses such as salt, oxidative stress, and cadmium ions as well as a defense response to bacteria.

Because NAD^+^ is used as a noncanonical initiating substrate, whether NAD^+^ caps can be added post-transcriptionally remains unknown (43). However, NAD^+^ decapping proteins have been discovered. A study showed that *Arabidopsis* AtDXO1, a single-copy ortholog of the mammalian DXO1, is capable of removing NAD^+^ from transcripts (225), suggesting the NAD^+^-capping is a regulatable modification of mRNAs. In the absence of DXO1, NAD^+^-capped mRNAs are processed into small RNAs by RNA-dependent RNA polymerase 6 (RDR6), further facilitating their turnover. Interestingly, in plant responses to abscisic acid (ABA), there is a global NAD^+^ decapping of ABA-responsive transcripts, promoting their stability. However, this process is independent of AtDXO1, suggesting that other NAD^+^-decapping enzymes may play a role.

It has recently been discovered that a prokaryotic Toll-interleukin 1 receptor (TIR) domain–containing protein, AbTir, can remove the nicotinamide (NAM) moiety from NAD^+^-capped mRNAs (deNAMing) both in vitro and in vivo (197). Interestingly, this activity is conserved in TIR domain–containing proteins in other organisms, such as archaea, raising the question of whether TIR-domain proteins in plants, known for their key roles as immune receptors and signal transducers, also contribute to the deNAMing activity of NAD^+^-capped mRNA.

There are several important questions regarding the function and regulation of NAD^+^ and other noncanonical-capped mRNAs. First, it is crucial to understand the relationship between the available pool of metabolites in different cellular compartments and the noncanonical-capped mRNAs, especially during stress conditions when significant shifts in metabolism and the oxidation/reduction status of cellular and organelle environments occur. Additionally, it is important to explore how NAD^+^-capped mRNAs are translated. Are there specific initiation factors that recognize the NAD^+^ cap to start translation? Moreover, during stress conditions, when global translation is altered, do these mRNAs undergo preferential translation? Finally, what are the functions of other noncanonical caps, and how do they impact cellular processes?

### 3.3. Kozak Sequence

First identified by Marilyn Kozak (103) in 1981, the Kozak sequence is a conserved nucleotide motif surrounding the start codon in eukaryotic mRNAs, critical for efficient translation initiation (Figure 1). In mammalian cells, the consensus sequence GCC(A/G)CCAUGG (where AUG is the start codon) is frequently observed (104). This sequence facilitates additional interactions with the ribosome, enhancing start codon recognition (166, 167). Mutations within the Kozak sequence can significantly affect translational efficiency to a 20-fold range, with A at position −3 and G at position +4 (with A in AUG as +1) being the most crucial (105).

In plants, the Kozak sequences exhibit both conservation and divergence compared to animal systems. Although the A at position −3 and the G at position +4 remain critical, plant-specific Kozak sequences often contain adenines at −4 to −1 positions, contributing significantly to high translation efficiency (63, 101). These differences likely reflect adaptations to plant-specific translational needs, such as stress responses and developmental regulations. A recent study reveals that the highly abundant A/GC-type Kozak sequence enhances translation initiation-to-elongation transition through HOT TEMPERATURE 3 (HOT3)/eIF5B1, which preferentially promotes the translation of photosynthesis-related nuclear-encoded transcripts (65). An intriguing question remains: Do Kozak sequences, either independently or in conjunction with mRNA secondary structures or modifications, participate in modulating dynamic translation initiation under stress conditions? Further exploration will provide us the answer.

### 3.4. Upstream AUGs and Upstream Open Reading Frames

It has become apparent that upstream AUGs (uAUGs) and uORFs are widely present across eukaryotic transcriptomes, with approximately 64% of mRNAs in humans, 60% in mice, 55% in *Drosophila*, 55% in *Arabidopsis*, and 53% in soybean (230) (Figure 1). Although uAUGs generally have a less favorable Kozak sequence context than main AUGs (mAUGs) (34, 228), emerging evidence from Ribo-Seq and mass spectrometry data reveals that uAUGs can initiate translation under both normal and stress conditions (79, 122, 145, 206). When a uORF is translated, it typically represses downstream mAUG translation by causing ribosome stalling or dissociation before reaching the mAUG (143). Notably, transcripts containing uORFs are often enriched in transcription factors and kinases (69, 99). Overexpression of just main ORFs (mORFs) of certain uORF-containing transcripts, such as TL1-BINDING FACTOR (TBF1), ARABIDOPSIS HOMEOBOX 1 (AtHB1), and BASIC LEUCINE-ZIPPER 11 (bZIP11), has been shown to cause deleterious growth phenotypes (157, 212), suggesting that uORFs are crucial for repressing downstream protein production under normal growth conditions. The identification and inhibitory roles of uORFs in plants are detailed in the review (185, 190, 207).

The peptides encoded by most uORFs are not conserved. However, approximately 120 conserved peptide uORFs (CPuORFs), grouped into 81 homology groups, have been identified in *Arabidopsis* (69). Among these CPuORFs, five have been shown to cause ribosome stalling through interactions between the nascent peptide and the ribosome exit channel (68, 181, 214). However, the mechanisms by which other small peptides inhibit translation remain unclear. In mammals, only a small subset of uORF-encoded peptides has been detected through mass spectrometry, likely because of technical limitations or the rapid degradation of these peptides immediately after their production (168). It remains uncertain whether these small peptides function in translation inhibition or other biological processes or whether their importance primarily lies in the translation initiation from their uAUG.

Recent studies have shown that the uORF-mediated inhibition can be alleviated in plants in response to various environmental stresses, enabling increased production of downstream proteins to facilitate fast adaptation. These stress conditions include pathogen infection (144, 208, 212) and hypoxia (92), environmental cues like light exposure (108) and elevated boron levels (176), and changes in nutrient and metabolite availability, such as phosphate (9), sucrose (154, 203), and polyamine (64) levels. These data demonstrate the role uORFs play as molecular switches in controlling the production of downstream proteins.

Such a regulatory mechanism has been applied to optimize gene expression in crops. For instance, incorporating the 5′ LS of TBF1 transcript containing two uORFs in front of the coding sequence of the immune regulator NPR1 enables pathogen-inducible translation of NPR1. As the application of uORFs in engineering crop performance becomes more common (139), further understanding of their regulatory mechanisms will be essential for allowing rational design of their application in crop improvement. For example, when multiple uORFs are sometimes present in a single transcript and/or interact with other translational elements, such as RNA secondary structures and modifications, AI-based computational tools will play a critical role in accurately predicting the activities of specific uORFs within a given 5′ LS and identify target sequences and strategies for manipulation.

### 3.5. mRNA Secondary Structures

mRNAs fold into diverse base-pairing conformations, forming secondary structures such as stems, loops, and bulges as well as tertiary structures like pseudoknots and RNA G-quadruplexes (RG4s), which are highly dynamic in cells (Figure 1). Recent advances in techniques for probing in vivo RNA secondary structures such as selective 2′-hydroxyl acylation analyzed by primer extension and mutational profiling (SHAPE-MaP) (169, 170), in vivo click-SHAPE (icSHAPE) (173), and dimethyl sulfate-MaPseq (DMS-MaPseq) (243) have greatly expanded our ability to identify and study the functions of these structural elements. mRNA structures are now recognized as key regulators of a wide range of biological processes, including transcription, translation, mRNA stability, localization, and condensate formation (reviewed in 6, 26, 117, 174). Here, we focus on the dynamic structural changes in mRNA that modulate translation in plants.

Similar to the findings in mammals and yeasts, transcriptome-wide and gene-specific studies in plants show that double-stranded mRNA structures (e.g., long hairpins and G-quadruplexes) upstream of the start codon generally inhibit translation initiation (110), and reducing the structural complexity in these regions enhances translation efficiency (41). Notably, the dynamic nature of these structures enables context-dependent regulation of translation. For example, translation of PHYTOCHROME-INTERACTING FACTOR 7 (PIF7), a homolog of PIF4 involved in temperature sensing, is regulated by a stable hairpin upstream of the start codon (37). Elevated temperatures destabilize this hairpin, leading to enhanced PIF7 translation. Similarly, high light conditions trigger the unwinding of a hairpin upstream of the start codon for the photosystem protein psbA (55). This conformational change, likely mediated by RNA-binding proteins, enhances psbA translation, facilitating efficient photosystem repair. Furthermore, the zinc-finger protein JULGI ( JUL) promotes the formation of G-quadruplexes in the 5′ LS of *SMXL4* and *SMXL5* mRNAs, suppressing their translation (35). This suppression is vital for proper phloem differentiation, as JUL deficiency leads to increased phloem cell formation and enhanced sink strength.

Interestingly, in contrast to hairpins upstream of the start codon, the structure located downstream of the start codon can facilitate translation initiation (61, 72, 191, 208). In *Arabidopsis*, immune-related transcripts are found to be enriched with uAUGs whose translation is enhanced by downstream hairpins with folding energies within a specific range (−19.9 kcal/mol to −34.1 kcal/mol) (208). Under normal conditions, these hairpins promote start codon recognition at the uAUGs by slowing and engaging the scanning preinitiation complex to start translation from uAUG instead of the downstream mAUG that encodes immune proteins, thereby preventing unnecessary immune activation during normal growth. Upon pathogen infection, there is an increased expression of RH37-like helicases, which is associated with the preinitiation complex to unwind the hairpins, enabling ribosomes to bypass the uAUGs to initiate translation at the mAUG to promote the immune protein production. This mechanism provides a rapid switch from growth to defense.

Beyond directly influencing ribosome scanning, plant mRNA structures regulate translation through additional mechanisms, including affecting mRNA stability (220), phase separation (234), and intracellular transport (232). Cold-adapted plant species tend to evolve guanine-enriched transcriptomes, promoting the formation of RG4s in response to low temperatures (220). These RG4s globally stabilize mRNAs, facilitating cold adaptation. In the mRNA of SHORT ROOT, an essential regulator of root development, RG4s drive the formation of phase-separated condensates (234), indicating a potential layer of translational control. Additionally, phloem-mobile mRNAs are enriched with tRNA-like structures containing a stem-bulge–stem-loop confirmation. These structures facilitate mRNA movement through phloem flow or pass through graft junctions, allowing them to be translated at distant sites (232).

Future research will focus on developing advanced techniques and algorithms to elucidate how mRNAs fold into complex tertiary structures and interact with other components [e.g., RNA binding proteins (RBPs) and plant cell metabolites] to regulate translation. A nanopore based single-molecule RNA structural probing technique has been developed (13) to uncover the dynamic nature of RNA in regulating various biological processes. Single-cell resolution RNA structural probing (193) and Ribo-Seq (183) will allow for the detection of mRNA structures and translation activities across different plant cell types. Advances in these new areas will deepen our understanding of how plants control mRNA structures to adapt translation in response to developmental and environmental changes.

### 3.6. mRNA Modifications

Recently developed techniques have enabled transcriptome-wide base-resolution detection of various RNA modifications in plants, including N^6^-methyladenosine (m^6^A), 5-methylcytosine (m^5^C), N^1^-methyladenine (m^1^A), and pseudouridine (Ψ), with their important roles in a variety of cellular processes being identified (reviewed in 152, 164) (Figure 1). Among these modifications, m^6^A stands out as the most abundant mRNA modification (201). This reversible modification is deposited by methyltransferases (writers, including METHYLTRANSFERASE A, METHYLTRANSFERASE B, FKBP INTERACTING PROTEIN 37, VIRILIZER, and others), recognized by m^6^A-binding proteins [readers, including the EVOLUTIONARILY CONSERVED C-TERMINAL REGION (ECT) family proteins], and removed by demethylases (erasers, including ALKBH-homologous proteins) (reviewed in 226). It regulates diverse molecular functions, including chromatin state, alternative polyadenylation, mRNA stability, translation, and microRNA (miRNA) maturation, and plays critical roles in various biological processes, such as responses to pathogen and virus infections and light, oxidative, heat, and high-salinity stresses (reviewed in 177).

Global profiling provides valuable insights into the overall impact of m^6^A on translation (31, 78, 126). However, m^6^A in different subsets of transcripts and at distinct locations in a transcript [e.g., 5′ LS, coding sequence, or 3′ untranslated region (3′ UTR)] may have diverse functions. Investigating the effects of m^6^A on translation through studies of writer mutants often proves challenging, as these mutants tend to exhibit pleiotropic effects, making it difficult to isolate the direct roles of m^6^A in translation. A more precise approach involves studying the function of writers or readers that target specific subsets of transcripts (31). This targeted analysis enables a finer dissection of the distinct roles of m^6^A modifications on individual transcripts. For example, a study found that although the overall m^6^A abundance in mRNA remained unchanged during PTI, m^6^A modifications were specifically enriched in immune-related transcripts (31). Even more intriguing, m^6^A plays a dual role in regulating these transcripts by enhancing their translation efficiency to ensure rapid immune protein production while promoting their fast turnover to resolve the immune response. These dual effects are mediated through the distinct binding of reader proteins. Although ECT2 and ECT3 enhance mRNA stability and translation, ECT1 promotes mRNA degradation in *Arabidopsis* (31). Similarly, overexpression of the ECT homolog in apple, MhYTP2, enhances the translation efficiency of its target transcripts, including encoding the antioxidation enzyme MdGDH1L, which leads to increased antioxidant activity and improved resistance to powdery mildew (62).

Despite the progress in understanding the function of m^6^A, much remains unknown about how RNA modifications, such as m^5^C, m^1^A, and Ψ, influence translation and other biological processes in plants. Future research should investigate possible writers, readers, and erasers for these modifications to pinpoint their specific roles in stress regulation. In addition, advances in single-molecule detection will further enable insights into how these modifications, working alone or in combinations, shape the fate and function of individual mRNAs.

### 3.7. Alternative Splicing

Alternative splicing (AS) mainly includes exon skipping, intron retention, and alternative 5′ and 3′ splice sites (reviewed in 128). The mRNA variants generated through AS, with the potential of insertions or deletions of start/stop codons, introns, or alterations of 5′ LS or 3′UTR, could affect translation efficiency and the protein product. AS is largely involved in plant responses to environmental cues (reviewed in 58, 113) (Figure 1).

In plants, intron retention is the most prevalent form of AS (89), and could regulate the timing of translation (15, 89) by storing pre-mRNAs in the nucleus and protecting them from degradation through nonsense-mediated decay until translation is required (15). A striking example is observed in the fern *Marsilea vestita* gametophytes, where transcripts encoding proteins essential for gamete development are retained as intron-containing forms during desiccation (15). Upon rehydration, a cascade of splicing regulators is activated to remove the introns, converting these pre-mRNAs into translationally active mRNAs. Similarly, in seedling photomorphogenesis, the E3 ubiquitin ligase CONSTITUTIVE PHOTOMORPHOGENIC1 (COP1) regulates light-signaling pathways by modulating spliceosome activity (238). COP1 binds to and ubiquitinates the spliceosome component DOMINANT COP1-6 SUPPRESSOR 1 (DCS1) to control intron retention in light-regulated transcripts and modulate their translation in response to light conditions.

AS also directly influences translation efficiency by altering the 5′ LS of transcripts. For example, a global spatial study found that splicing of the 5′ LS in LATE ELONGATED HYPOCOTYL transcript (*LHY*, encodes a key circadian clock regulator) varies among *Arabidopsis* haplotypes distributed in different temperature regimes (86, 160). These temperature-dependent splicing variants exhibit distinct sequences and structures in their 5′ LSs, some of which contain uAUGs while others lack them, thereby modulating translation initiation (86). In another case, light-induced intron retention in the transcript of PHYTOCHROME-INTERACTING FACTOR 3 (PIF3) introduces a uAUG in its 5′ LS (45). The resulting uORF inhibits the translation of PIF3, fine-tuning protein levels and facilitating oscillations in PIF3 expression during short-day light cycles.

Emerging evidence in the past decade highlights the key function of AS in regulating gene expression under abiotic stresses, including heat, drought, and high salinity (reviewed in 113). However, its role in plant immune responses remains largely unexplored. Intriguingly, during PTI, certain pattern recognition receptors, such as FLAGELLIN-SENSITIVE 2, Nt-Sd-RLK, BRI1-ASSOCIATED RECEPTOR KINASE 1, and CHITIN ELICITOR RECEPTOR KINASE 1, undergo AS (reviewed in 58), suggesting a dynamic role for splicing in regulating immune receptor production. A recent global profiling of AS events during plant PTI reveals significant changes in splicing patterns of immune-related transcripts, largely regulated by RNA polymerase II C-terminal domain phosphatase-like 3 (CPL3) (100). Altogether, understanding how AS modulates these immune components will deepen our comprehension of plant defense strategies.

### 3.8. Small RNAs

Small RNAs, such as miRNAs and small interfering RNAs (siRNAs), play crucial roles in gene regulation by forming the RNA-induced silencing complex (RISC) upon binding with ARGONAUTE proteins (16). Upon binding to the mRNA target, RISC typically mediates mRNA cleavage or translation inhibition (16, 21) (Figure 1). An in vitro study suggests that RISC inhibits mRNA translation by directly blocking the recruitment or scanning of the ribosomes (84). In plants, miRNA-mediated translational inhibition occurs on the endoplasmic reticulum (ER), a process requiring the ER-associated protein ALTERED MERISTEM PROGRAM1 (AMP1) (118). Unlike mRNA cleavage, translation inhibition is AMP1-dependent, as AMP1 interacts with ARGONAUTE1 (AGO1) to prevent miRNA-targeted transcripts from being recruited to the ER membrane–bound polysomes. Furthermore, recent findings show that another ER-localized protein, HYPONASTIC LEAVES1, interacts with AGO1, AMP1, and the target mRNAs to repress their translation (218).

Besides miRNA, recent studies reveal that 22-nucleotide (nt) siRNAs in plants can also inhibit translation (204). A group of 22-nt siRNAs is massively produced, particularly derived from two nitrate reductase genes, NITRATE REDUCTASE 1 (NIA1) and NITRATE REDUCTASE 2 (NIA2), in the mutant line deficient in cytoplasmic RNA decay and DCL4 activity. Unlike 21- and 24-nt siRNAs, these 22-nt siRNAs are less effective at target cleavage but mainly function in translational repression. Notably, NIA1/NIA2-derived 22-nt siRNAs are preferentially generated under environmental stresses such as nitrogen deficiency, ABA treatment, and high salinity to inhibit translation and facilitate a shift from growth to stress response.

In addition to miRNAs and siRNAs, tRNA-derived fragments (tRFs) have emerged as modulators for post-transcriptional gene silencing and translational regulation in plants (reviewed in 127, 147). The abundance of tRFs is dynamically modulated by various stresses, including oxidization stress, heat, drought, high salinity, UV stress, and phosphate deficiency, as well as pathogen attacks (reviewed in 127, 147), highlighting their potential roles in modulating global response to environmental cues. Recent studies revealed that when incorporated into AGO1-associated RNA-induced silencing complexes, tRFs facilitate mRNA cleavage (130, 155). This process can be exploited by pathogenic tRFs to hijack the host silencing machinery, suppressing critical host transcripts (155). Additionally, in vitro experiments revealed that tRFs can inhibit translation by directly associating with polysomes (112), with specific tRFs derived from tRNA^Ala^ (AGC) and tRNA^Asn^(GUU) showing particularly strong translation attenuation. However, the mechanisms underlying tRF association with active polyribosomes and the broader impact of tRF population dynamics on protein synthesis during stress responses remain to be elucidated.

## 4. TRANSLATIONAL REGULATION THROUGH RIBOSOMAL BIOGENESIS AND HETEROGENEITY

The eukaryotic 80S ribosome is a complex molecular machine comprising a small 40S subunit and a large 60S subunit, assembled from approximately 81 ribosomal proteins (RPs) and four ribosomal RNAs (rRNAs): 25S (28S in mammals), 18S, 5.8S, and 5S (23). Ribosome biogenesis starts in the nucleolus with the transcription of the 45S precursor rRNA (pre-rRNA) from rDNA (reviewed in 7, 159, 202). This pre-rRNA is then processed into mature rRNAs through splicing, modifications, and folding, facilitated by various ribonucleoproteins (RNPs). Meanwhile, RPs are synthesized in the cytoplasm and imported into the nucleolus to participate in the assembly process. In the nucleolus, the 18S rRNA assembles with RPs to form the precursor 40S ribosomal subunit (pre-40S), whereas the 25S rRNAs and 5.8S, along with the separately transcribed 5S rRNA, combine with RPs to produce the precursor 60S subunit (pre-60S). These immature ribosomal subunits are then transported to the nucleoplasm, where they undergo additional maturation steps before being exported to the cytoplasm. Final assembly and maturation of the ribosomal subunits occur in the cytoplasm, yielding functional ribosomes.

Given its central role in translation, regulation of ribosome biogenesis is considered a key step during stress response in plants (Figure 1). This frequently involves upstream signaling pathways, mediated by central regulators such as TOR and phosphorylating S6 kinase (S6K) (see below), to modulate ribosomal components (such as rRNA sequences, RP composition, and modifications in rRNAs or RPs), resulting in ribosome heterogeneity (reviewed in 56, 162). Notably, genome duplication events in *Arabidopsis thaliana* have resulted in multiple RP paralogs, with two to seven paralogs per RP gene (23), allowing for adaptive translation under different conditions.

Using transcriptomics, polysome profiling, and proteomics approaches, new studies have found changes in the abundance or post-translational modifications of RPs under stress conditions, such as heat, UV, phosphate deficiency, and immune responses (summarized in 131). However, the functional consequences of these changes, particularly the impact on the translational efficiencies of global or a subset of transcripts, remain largely unexplored. The essential roles of ribosomal components in plant growth make it challenging to disentangle their functions during stress responses. Studies using transient silencing approaches have shed light on their specific roles. For instance, using virus-induced gene silencing of RPs such as RPSaA, RPS5A, and RPS24A has demonstrated their roles in translating defense-related proteins, including catalase and peroxidase (51).

In addition to RP heterogeneity, emerging evidence shows that stress conditions can also influence pre-rRNA processing in plants, leading to heterogenized rRNAs (summarized in 159). Pre-rRNA processing occurs in the nucleolus, which is a membraneless biomolecular condensate that exhibits changes in morphology and function during stress conditions and is frequently targeted by pathogen effectors during infections (reviewed in 114). This raises the interesting question about whether and how stress-induced alterations in nucleolar dynamics affect rRNA heterogeneity and, consequently, ribosome behaviors.

Another layer of regulation involves the nuclear transport of pre-ribosomes. Recent studies have shown that *Arabidopsis* ARGININE METHYLTRANSFERASE 3 (AtPRMT3), an evolutionarily conserved arginine methyltransferase, is critical for pre-rRNA processing and facilitates ribosome biogenesis through its interaction with the RP RPS2B. The loss-of-function mutant *atprmt3* displays nucleolar stress and pleiotropic growth defects (66, 67). Notably, these defects can be rescued by mutation of PROGRAMMED CELL DEATH 2 (PDCD2), a zinc finger protein that shuttles between the nucleus and cytoplasm through the nuclear pore complex (NPC) to mediate pre-ribosome export (199). Intriguingly, the AtPRMT3-RPS2B complex has been shown to balance growth and stress responses by promoting the translation of housekeeping genes while repressing cold-responsive mRNAs; dysfunction of this complex enhances plant freezing tolerance (199). These studies provide a mechanistic link between ribosomal biogenesis, pre-ribosome transport, and plant stress adaptation.

Moving forward, several key questions still remain: What is the mechanism underlying stress-induced ribosomes in preferentially targeting a subset of mRNAs? How do heterogeneous ribosomes, differentially localized within the cell, influence translation? Are there cell-type-specific ribosomes in plants, and what roles do they play? Advances such as single-particle cryo-EM (140) and single-particle mass spectrometry (111) will enable the characterization of ribosomal compositions at the molecular level. High-resolution live-cell imaging (59) and single-molecule tracking (77) will facilitate real-time observations of ribosome movements. Along with the rapid development of single-molecule high-resolution RNA modification sequencing (115), these new techniques will largely enhance our understanding of how ribosome biogenesis and heterogeneity regulate translation during stress.

## 5. tRNA- AND CODON-DEPENDENT TRANSLATIONAL REGULATION

During protein synthesis, decoding by tRNAs is a crucial and complicated process that assures both the efficiency and accuracy of protein production (Figure 1). This process involves the biogenesis of tRNAs, which starts from the transcription of pre-tRNA in the nucleus, followed by tRNA processing, including leader and tailer sequence removal, the addition of the 3′ CCA tail, folding, and modifications that enhance tRNA stability and codon recognition (reviewed in 11). The successful export of mature tRNAs via the NPC to the cytoplasm, along with their repair and turnover, ensures that only functional tRNAs participate in translation. In the cytoplasm, tRNA aminoacylation is catalyzed by aminoacyl-tRNA synthetases, which attach amino acids to tRNAs in a process regulated by amino acid availability and the synthetase activity. Additionally, codon bias in mRNAs significantly affects translation, as frequently used codons that match abundant tRNAs improve efficiency, whereas rare codons may slow translation and lead to mRNA decay.Developmental cues and stress conditions can further regulate tRNA populations and codon usage, enabling plants to preferentially translate certain proteins under given conditions.

Plant tRNA biogenesis involves multiple layers of regulation, and the underlying mechanisms remain largely unknown. This is largely due to the challenges in developing tRNA sequencing techniques capable of unbiasedly quantifying the sequence, the expression levels, and the modification stoichiometry of full-length tRNAs, their precursors, and their turnover products, all of which are critical for understanding the dynamic regulation of tRNA biogenesis. Development of new techniques in plants such as tRNA-seq (200) and FINE-tRNA-seq (215) has significantly advanced our understanding of this regulation.

Transcription of tRNAs is tightly regulated during various abiotic and biotic stresses. In eukaryotes, the transcription of tRNAs, 5S rRNAs, and certain small RNAs is carried out by RNA polymerase III (Pol III) (5). A key regulator of Pol III activity is MAF1, a conserved suppressor of Pol III in eukaryotes, which is directly controlled by TOR kinase through phosphorylation under various stress conditions (to be discussed in detail below), underlying a pivotal mechanism linking tRNA and ribosome biogenesis to TOR-mediated plant stress responses (1).

Besides tRNA levels, emerging methods for quantifying tRNA modifications have shed light on how these modifications maintain tRNA stability, translation efficiency, and proper aminoacylation. Among these, tRNA thiolation, an evolutionarily conserved modification, is essential for plant immunity in *Arabidopsis* (236). Loss of tRNA thiolation enzyme ROL5 results in disrupted transcriptome and proteome reprogramming during immune responses, including reduced translation of the salicylic acid (SA) receptor NPR1, leading to impaired SA signaling and hypersusceptibility to *Pseudomonas syringae*, underscoring a critical role of tRNA thiolation in translation and immune regulation in plants (236). Similarly, C^2^-methyladenosine at tRNA position 37 (m^2^A37), installed by the methyltransferases RlmN-like proteins (RLMNL1, RLMNL2, and RLMNL3), is present in both cytosolic and chloroplast tRNAs in plants (47). m^2^A37 enhances translation efficiency by facilitating the decoding of tandem m^2^A-tRNA-dependent codons (CAA, CAG, CGU, and CGC), and the *rlmnl1*, *rlmnl2*, and *rlmnl3* mutants show hypersensitivity to the aminoglycoside class of antibiotics and increased resistance to osmotic stress. Additionally, the tRNA^His^ guanylyltransferase ADAPTATION TO ENVIRONMENTAL TEMPERATURE 1 (AET1) is important for plant growth and survival during heat stress (30). AET1 maintains tRNA homeostasis by adding a guanosine to the 5′ end of pre-tRNA^His^. During heat stress, AET1 supports plant survival by enhancing the translation of heat-responsive uORF-containing mRNAs, such as OsARF19 and OsARF23. This is achieved through its interaction with RACK1A and eIF3h, which are required for the translation reinitiation of uORF-containing transcripts (161). In the rice blast pathogen *Magnaporthe oryzae*, N^1^-methyladenosine at tRNA position 58 (m^1^A58), catalyzed by the tRNA methyltransferase Trm6/Trm61 complex, enhances translation elongation rates by facilitating eEF1–tRNA binding, and its absence drastically impairs translation efficiency and consequently fungal virulence, presenting a promising target for controlling rice blast (71). Together, these studies highlight the diverse and critical roles of tRNA modifications in translational regulation during stress responses.

Numerous studies have revealed that amino acid metabolism is altered under various stress conditions (52, 73, 138). As amino acid availability is crucial for tRNA aminoacylation, this raises intriguing questions about whether tRNA aminoacylation status is altered during stress and how these changes affect translation. Recent progress in sequencing-based methods has enabled the quantification of aminoacylated versus uncharged tRNAs, revealing the tRNA aminoacylation landscape in *Arabidopsis* (28). This study is just the beginning, as it remains fascinating to explore the global tRNA aminoacylation dynamics in response to stress conditions and their impact on translational outcomes.

In addition to tRNA synthesis, modification, and aminoacylation, tRNA repair is another critical step in plant response to changing conditions. In rice, THERMO-SENSITIVE GENIC MALE STERILE 5 (TMS5) encodes a conserved 2′,3′-cyclic phosphatase whose mutation accounts for 95% of thermosensitive genic male sterility (TGMS) lines (215). A recent study found that at high temperatures, the *tms5* mutant fails to repair damaged cP-ΔCCA-tRNAs as part of the ribosome-associated quality control, preventing the addition of the CCA tail and the production of mature tRNAs, ultimately resulting in male sterility (215). This discovery reveals a novel mechanism for the regulation of tRNA biogenesis under stress conditions, offering valuable insights for improving TGMS in hybrid crop development.

Moving forward, it will be important to explore how the decoder tRNA and the decoding machinery ribosome, in conjunction with the mRNA, collectively regulate translation elongation under various conditions. Recent advances in probing ribosome behavior, including modification (e.g., ubiquitination and phosphorylation), stalling, collision, and quality control (reviewed in 221), at both the cellular level and single-mRNA level will provide exciting avenues for understanding these dynamic processes. Structural insights into ribosome–tRNA interactions will provide detailed molecular-level insights into how tRNAs are accurately selected and positioned during decoding, how specific tRNA modifications influence codon recognition and translation efficiency, and how tRNA mischarging, mispairing, or depletion would cause ribosome stalling (90). Moreover, the continuous development of unbiased high-resolution tRNA probing techniques and the improved methods for detecting global metabolites, particularly those essential for tRNA synthesis, modification, aminoacylation, and repair, will significantly enhance our ability to map the dynamic tRNA landscape, uncovering the interplay between tRNA function and cellular metabolism. Advancements in the study of these processes, along with a deeper understanding of codon usage in mRNAs to decipher the demand-and-supply balance between codons and their corresponding tRNAs, could reveal mechanisms underlying selective translation of specific transcripts under stress conditions.

## 6. REGULATION THROUGH MASTER TRANSLATIONAL REGULATORS AND TRANSLATION INITIATION FACTORS

### 6.1. Target of Rapamycin

The TOR kinase plays a central role in regulating translation by integrating environmental and cellular cues such as light, carbon and nitrogen availability, amino acid levels, oxygen, temperature, stress signals, hormones, and energy status in plants (reviewed in 25). TOR promotes translation initiation by modifying S6K, which in turn phosphorylates translation-related proteins, including the RPs eS6 and eIF3h. The latter is involved in translation reinitiation of uORF-containing transcripts (161). In addition, TOR enhances ribosome biogenesis by regulating the translation of RP mRNAs containing 5′ terminal oligopyrimidine (5′ TOP) motifs and facilitates both ribosome and tRNA biogenesis by phosphorylating and inhibiting MAF1, a key repressor of RNA polymerase III activity. In mammals, TOR also regulates translation initiation through phosphorylating 4E-binding protein (4E-BP), preventing it from binding to eIF4E (57), thereby enabling eIF4E to interact with eIF4G and form the eIF4F complex to initiate translation. However, homologous 4E-BPs are absent in plants, although nonhomologous 4E-BP-like proteins (4EBP1/4EBP2) that perform similar functions to mammalian 4E-BP have recently been identified (46).

### 6.2. Sucrose Non-Fermenting-1-Related Protein Kinase 1

Although TOR mainly promotes plant growth and translation under favorable conditions, SnRK1 acts as a stress and energy sensor, repressing TOR activity under stress or during low energy states (reviewed in 22). SnRK1 directly regulates translation by phosphorylating key components such as eIF4E and eIF(iso)4G1, leading to global translational repression while enabling the selective translation of stress-responsive mRNAs, such as those involved in hypoxia tolerance (36).

### 6.3. General Control Nonderepressible 2

It has been shown that GCN2 plays an important role in plant translational control by phosphorylating eIF2α under certain stress conditions such as amino acid depletion (233). In yeast and mammals, GCN2 not only suppresses general protein synthesis but also promotes the selective translation of uORF-containing stress-responsive mRNAs. The well-studied examples include yeast GENERAL CONTROL NONDEREPRESSIBLE 4 (GCN4) (75) and mammalian ACTIVATING TRANSCRIPTION FACTOR 4 (ATF4) (184), whose uORFs inhibit their translation under normal conditions. During stress, GCN2-dependent eIF2α phosphorylation results in reduced translation initiation from the uAUG and prolonged scanning of the preinitiation complex to translate the downstream mORFs to promote cell survival. However, in plants, this integrated stress response pathway appears less central to most biotic and abiotic stress responses (reviewed and discussed in 125). Instead, plants have evolved alternative mechanisms for selectively translating stress-related transcripts during adaptation to environmental challenges (17, 30, 97, 192, 199, 208, 211). Unlike animals that possess highly specialized immune cells, plant cells are multifunctional; thus, stress response mainly involves translational reprogramming to prioritize the synthesis of stress-related proteins critical for adaptation instead of globally shutting down translation. In plants, GCN2-mediated phosphorylation of eIF2α is not tightly coupled to translational repression, likely because of the reduced dependence on eIF2B-mediated GDP–GTP exchange, unlike in animals and yeast (237). Thus, the role of GCN2 and eIF2α in stress regulation needs further investigation.

### 6.4. eIF2

In contrast to eIF2 phosphorylation by GCN2 in response to amino acid depletion and hypoxia, plants utilize a distinct mechanism to induce effector-triggered immunity (ETI)-associated translation. During ETI, elevated ATP levels activate the ATP-grasp protein CDC123, which facilitates the eIF2 complex assembly to induce the global defense translatome (32).

### 6.5. eIF4F and eIF(iso)4F

A distinctive feature of the plant translation initiation complex is the presence of two alternative complexes: the eIF4F complex (with eIF4E and eIF4G) and the eIF(iso)4F complex [composed of eIF(iso)4E and eIF(iso)4G] (137). These gene duplication products have provided plants with the flexibility to regulate the translation of specific subsets of mRNA under certain conditions.

Both eIF4E and eIF(iso)4E function as cap-binding proteins; however, the structural differences result in eIF(iso)4E having a lower binding affinity for the 5′ m^7^G cap than eIF4E (106). The difference in cap-binding affinity has led to their different translation regulatory features. During infection, plant RNA viruses lacking a canonical 5′ cap structure exploit the host translation machinery by hijacking eIF4E and/or eIF(iso)4E to facilitate the translation of their viral mRNAs. Interestingly, the binding affinities of different viral mRNAs/genomes to eIF4E and eIF(iso)4E vary, with some viruses showing a preference for one over the other (241). To counteract viral infection, plants have evolved natural resistant alleles with mutations in eIF4E or eIF(iso)4E that disrupt viral interactions, effectively preventing viral replication, while maintaining their essential roles in host cellular translation (8). Such mutations have become important tools in breeding for improving crop resistance to viral pathogens.

Most eukaryotes contain two eIF4G isoforms that share high sequence and structural similarities. However, plant eIF4G and eIF(iso)4G are notably different in size and domain organization, with eIF(iso)4G being significantly shorter than eIF4G (53). Both isoforms interact with eIF4B, eIF4A, and PABP, but eIF4G has two binding domains for each of these partners, whereas eIF(iso)4G has only one for eIF4B and PABP (33, 53). The different features of the two isoforms suggest their distinct functions in translational regulation. As discussed above, pathogen challenge leads to the phosphorylation that inhibits eIF4G activity while promoting eIF(iso)4G-driven translation of defense-related mRNAs (192).

## 7. SPATIAL DYNAMICS OF TRANSLATION

### 7.1. mRNA transport

The journey of mature mRNAs as they are exported through the nuclear pore may lead to different subcellular destinations for translation. The nuclear export of mRNAs is a highly conserved pathway across eukaryotes (Figure 2). Eukaryotic mRNA is synthesized in the nucleoplasm by RNA polymerase II (Pol II) and undergoes essential processing, including 5′ capping, splicing, and 3′ polyadenylation (10). During this process, nascent mRNAs are associated with RBPs to form messenger RNPs (mRNPs), which facilitate the regulation of mRNA biogenesis, maturation, and export (10). A key component of mRNP is the transcription-export (TREX) complex, composed of the THO core complex and the associated proteins, including the RNA helicase U2AF associated protein 56 (UAP56) and the mRNA transport adaptors ALY/REF EXPORT FACTOR and UAP56-INTERACTING EXPORT FACTOR (homologs of human UAP56-INTERACTING FACTOR) (reviewed in 48, 163). In mammals and yeast, mRNA transport adaptors recruit exporters such as NXF1-NXT1 (in mammals) and Mex67-Mtr2 (in yeast), which directly bind to the NPC components to facilitate the export of mRNPs into the cytoplasm (163). However, the homologous mRNA exporters in plants have yet to be uncovered. Once in the cytoplasm, the mRNP disassembles, and some of its components are removed from the mRNA, guided by the DEAD-box helicase LOW EXPRESSION OF OSMOTICALLY RESPONSIVE GENE (homologous to human DBP5) and GLFG LETHAL 1, which are located on the cytoplasmic side of the NPC (48, 163). This results in a remodeled mRNP, preparing the mRNA for its next functional stage in the cellular process such as immediate translation or storage in granules for translation at a more suitable time.

Under stress, global mRNA export is impaired through posttranslational modifications or phase separation of mRNP components (142). This leads to nuclear retention of most mRNAs, yet stress-specific mRNAs are selectively exported. In animals, this selective export is facilitated by stress-specific transport adaptors to enhance transport efficiency mediated by specialized NPC components or by downregulation of quality-control mechanisms to prioritize the rapid export of stress-responsive transcripts (reviewed in 163). In plants, stress conditions such as PTI and hypoxia result in global translational reprogramming to prioritize stress-related mRNA translation (17, 211). Whether nuclear export contributes to this selective translation remains unclear and requires further investigation.

With the rapid development of high-resolution imaging techniques, studies across all kingdoms of life have revealed that mRNAs can be transported to specific cellular compartments for translation, enabling spatial regulation of protein synthesis. The most common mechanism for this involves active mRNA transport, where mRNA is actively directed to specific locations for translation by RBPs within the mRNP complex, rather than passive diffusion (reviewed in 39, 179). In this process, RBPs recruit motor proteins, such as kinesin, dynein, or myosin, to shuttle mRNAs along microtubules or actin filaments to specific cellular locations (39). Many mRNAs contain sequence elements, or sometimes secondary structural elements, referred to as zipcodes. These zipcodes are typically found in the 3′ UTR but can also be detected in the 5′ LS or the coding region. Each zipcode can be recognized by specific RBPs, which bind to and direct the mRNA to its target location (39). For example, in rice endosperm cells, mRNAs encoding the storage proteins prolamin and glutelin are transported to distinct ER subdomains guided by the zipcodes in the 3′ UTR and their associated RBPs, ensuring proper protein sorting and transport (120, 180). Similarly, in *Arabidopsis*, the mRNA for the VOLTAGE-DEPENDENT ION CHANNEL is transported to mitochondria via zipcodes in its 3′ UTR (136). In *Arabidopsis* root hair cells, the tip-localized RAPID ALKALINIZATION FACTOR 1–FERONIA complex facilitates the polarized localization of the translation initiation factor eIF4E1 and phosphorylates it, enhancing its binding affinity to mRNAs necessary for root development, suggesting spatial regulation of translation during root growth (240). These findings raise important questions about how widespread spatial translational regulation is across eukaryotes and whether it is involved in stress responses that have spatial specificities, such as at the penetration sites of fungal haustoria or root hairs that form infection threads triggered by nitrogen-fixing bacteria. With the development of new techniques like single-molecule fluorescence in situ hybridization (235) and other mRNA visualization tools, future research will uncover new insights into this intriguing area of study.

mRNAs can be transported for translation not only within individual cells but also to neighboring cells via plasmodesmata and over long distances through the phloem vascular system (reviewed in 102). These transported mRNAs are involved in biological processes such as cell fate determination, tuber formation, flowering, and nutrient allocation (102). Interestingly, plant mRNAs can be packaged into extracellular vesicles and transported into pathogen cells during infection (194, 195) (Figure 2). These mRNAs are then translated using the pathogen’s translation machinery to produce proteins that disrupt pathogen functions, such as impairing mitochondrial activity. In the reversed direction, pathogens can also deliver their RNAs into plant cells to dampen host immunity during infection (70), highlighting an evolutionary arms race between pathogens and their plant hosts through delivery of RNAs as weapons.

### 7.2. Biomolecular Condensates

Apart from transporting mRNAs to specific locations for translation, mRNAs can also be sequestered in membraneless biomolecular condensates like SGs and P-bodies (Figure 2). SGs are stress-inducible condensates composed of translationally stalled mRNAs, various translation initiation factors, RBPs, ATPases, and other proteins (85, 153). In plants, SGs form in response to heat stress, osmotic stress, high salinity, low oxygen, and immune challenges, and they typically disassemble once the stress subsides (reviewed in 49, 50, 149, 196). The molecular function of SGs mainly involves temporary storage for untranslated mRNAs, allowing plants to shut down translation and then rapidly resume it when conditions improve (172, 210, 239). For example, during plant ETI, a process involving programmed cell-death of infected tissue, hematopoietic protein-1 (HEM1) limits excessive cell death by forming condensates through its low-complexity domain (LCD) to sequester and inhibit the translational machinery, thus repressing translation of prodeath immune transcripts (239). Similarly, phenolic acids (PAs), including SA, can be sensed by the plant SG marker protein RNA-BINDING PROTEIN 47B (RBP47B), which forms condensates with RPs to trigger global translation shutdown (209). In natural or agricultural ecosystems, this RBP47-dependent translational control allows plants to sense PAs released by neighboring species and respond to their allelopathic effects. Plant SG formation is a highly dynamic and reversible process. During heat stress, the β subunit of ADAPTOR PROTEIN (AP)-3 complex is rapidly recruited to the SGs, whereas during heat recovery, it facilitates the recruitment of the 19S regulatory particle to SGs to deubiquitylate SG components, a key step during SG disassembly which allows for the translation to resume and plant growth to recover (146).

In contrast to SGs, P-bodies are constitutively present and primarily associated with mRNA decay pathways, including decapping and degradation, while also serving as sites for mRNA storage (reviewed in 98). During stress, SG and P-body activities are highly dynamic and can exchange protein components (24). For example, P-bodies undergo rapid disassembly and assembly during plant PTI (224). The mRNA decapping enzyme DECAPPING 1 (DCP1), a key component of P-bodies, is phosphorylated by MPK3 and 6, leading to P-body disassembly. The phosphorylated DCP1 also exhibits enhanced association with EXORIBONUCLEASE 4 (XRN4), facilitating the degradation of immune-related transcripts, especially those encoding negative immune regulators (224). Together, these biomolecular condensates maintain a balance between preserving mRNA for future uses and degrading unnecessary transcripts, highlighting their intricate roles in stress response and translational control. Further research is needed to fully understand their individual molecular functions and their interplay in these processes.

## 8. TOOLS FOR STUDYING TRANSLATIONAL REGULATION

The past decade has witnessed significant innovations in techniques for quantifying translation dynamics both globally and at the single-nucleotide level. The toolkits available for studying translation in plants have been reviewed in Reference 133. Here, we focus on Ribo-Seq because it is the most game-changing technology.

Ribo-Seq is a sequencing-based method that provides a global view of ribosome occupancy on mRNAs. By treating mRNAs with ribonucleases, ribosome-protected fragments (ribosome footprints), typically 28–30 nucleotides in length, are isolated and sequenced to quantify ribosome occupancy across the transcriptome at base-level precision (82). This method enables the studies to (*a*) measure the translation efficiency of individual transcripts and analyze translation dynamics in response to changing conditions; (*b*) identify novel ORFs, including small ORFs (sORFs); (*c*) detect alternative or noncanonical translation start and stop codons; (*d*) identify ribosome stalling at specific codons; and (*e*) determine whether noncoding RNAs, such as long noncoding RNAs (lncRNAs) or circular RNAs, exhibit translational activity. Ribo-Seq has been used to study translational dynamics in plants under various changing conditions, including hypoxia (92), phosphate deficiency (9), drought (116), salt stress (219), hormone signaling (135), and immune responses (208, 211, 222). However, several limitations of this technique need to be considered when designing experiments: (*a*) Ribo-Seq reflects the ribosome position on mRNAs, which could be a result of active translation or ribosome stalling; (*b*) the short ribosome footprints make it challenging to map to genomes that have undergone recent duplications; and (*c*) technical challenges, such as optimizing digestion conditions and reducing rRNA contamination, make it costly to generate high-quality, high-depth data sets. Reducing the cost, simplifying the method, and standardizing the data analysis algorithms are required for wider and more high-throughput applications.

Emerging breakthroughs in techniques, such as single-particle cryo-EM (140), single-particle mass spectrometry (111), and single-molecule fluorescence microscopy (189), have enabled the study of translation at unprecedented resolution, allowing for the tracking of translation at individual ribosome and mRNA levels. Advances like single-cell RNA sequencing (scRNA-seq) (81) and ribosome profiling (scRibo-Seq) (183), spatial single-cell translatomics (RIBOmap) (227), translating ribosome affinity purification (TRAP) (93), and live-cell RNA imaging (244) have significantly improved our understanding of translational regulation in different cell types and cellular compartments.

## 9. ENGINEERING CROP STRESS RESISTANCE

Recent achievements in understanding translation regulatory mechanisms have made it possible to apply the knowledge gained for more precise engineering of crops with desirable traits using transgenic or gene-editing technologies. One successful example involved the use of uORFs and cellular IRESs. By placing the master immune regulator NPR1 downstream of the 5′ LS of TBF1 containing uORFs and R-motifs, NPR1 translation can be made pathogen-inducible, enhancing broad-spectrum disease resistance in rice while maintaining normal yield (212). uORFs have also been used in controlling endogenous genes through gene-editing technologies (2, 88, 119, 121, 124, 165, 178, 213, 217, 229). For example, base editing was used to introduce a series of uORFs with varying inhibitory strengths into the 5′ LS of the rice DWARF AND LOW-TILLERING transcript, which encodes a protein in the GRAS family involved in the brassinosteroid signaling pathway (213). This approach successfully generated rice plants with diverse heights and tiller numbers. Additionally, CRISPR technology has enabled the creation of a pool of uORF variants and allowed the high-throughput screening of functional uORFs in rice, leading to the identification and editing of the uORF of the plastid GLUTAMINE SYNTHETASE 2 gene, achieving broad-spectrum disease resistance with minimal yield penalty (178).

Engineering stress resistance using translation regulatory elements also requires a comprehensive understanding of the relationships among different translational regulatory elements. Applying advanced computational tools, particularly AI-based techniques, can revolutionize our ability to predict and optimize translational outcomes for gene editing. For example, by designing machine-learning algorithms based on RNA sequences and secondary structures, one can accurately predict translational initiation sites and determine the optimal features of translational regulatory elements (208). A recent breakthrough in this field is the development of a high-performance and interpretable RNA foundation model (FM) for plants, PlantRNA-FM, which integrates RNA sequence and structural features from 1,124 plant species (223). This model enables transcriptome-wide identification of translation regulatory elements, providing new possibilities for uncovering RNA functional motifs. The rapid evolution of these tools can greatly aid in optimizing the placement of regulatory elements and simulating gene expression dynamics under various stress conditions, ultimately significantly enhancing the efficiency and reliability of engineering stress-resistant crops.

## 10. CONCLUSION

The rapid advancements in technology for studying translation, coupled with the groundbreaking success in mRNA vaccines against COVID-19 through RNA modifications (95), have propelled this field to the forefront of life sciences. Its immense significance in fundamental biology as well as in a wide range of potential applications has gained more recognition. We can now investigate the impact of many regulatory steps in translation one at a time. However, predicting translational activities of mRNAs will require integration of information on all the regulatory elements and factors detailed above and more. The key to addressing this challenge lies in the broader application of AI, with the recently developed PlantRNA-FM as a great start toward rational design of protein production for engineering new crop species with resilience against pathogen challenge and abiotic stress while maintaining high yield. Inspired by breakthroughs like AlphaFold in protein structure prediction (91), we recognize that achieving our ambitious goal will take years of data collection and analysis. However, with the rapid progress in both biological research and AI, there is every reason to remain optimistic.
